# Perinatal BPAF Exposure Reprograms Offspring’s Immune–Metabolic Axis: A Multi-Omics Investigation of Intergenerational Hepatotoxicity

**DOI:** 10.3390/toxics14010097

**Published:** 2026-01-21

**Authors:** Shengjun Bai, Xiaorong Wu, Wei Mao, Mengan Guo, Yufeng Qin, Guizhen Du

**Affiliations:** 1 Key Laboratory of Modern Toxicology of Ministry of Education, School of Public Health, Nanjing Medical University, Nanjing 210029, China; sjbai@njfu.edu.cn (S.B.); gma0736@163.com (M.G.); 2 Department of Microbiology and Infection, School of Public Health, Nanjing Medical University, Nanjing 210029, China; 3 School of Public Health, Southwest Medical University, Luzhou 646099, China; xrong_wu@163.com; 4 Department of Special Clinic, Shanghai Children’s Hospital, School of Medicine, Shanghai Jiao Tong University, Shanghai 200240, China; weimao0619@gmail.com

**Keywords:** bisphenol AF, perinatal exposure, hepatic steatosis, multi-omics

## Abstract

Bisphenol AF (BPAF), a prevalent bisphenol A (BPA) substitute, raises concerns due to its environmental persistence and endocrine-disrupting potency. While metabolic effects of direct exposure are documented, its intergenerational consequences remain unclear. Here, we demonstrated that perinatal BPAF exposure induced persistent metabolic syndrome in offspring, including glucose intolerance, hepatic steatosis, and adipose hypotrophy. Integrating multi-omics data, we observed that BPAF exposure shaped offspring’s hepatic epigenome, as demonstrated by genome-wide alterations in H3K27ac-marked regulatory elements. This epigenetic rewiring indicated a dual regulatory effect on transcriptomes that suppressed interferon-γ responses while activating sterol biosynthesis, ultimately perturbating hepatic metabolome, including depleted pantothenate levels and accumulation of pro-inflammatory eicosanoids. Our findings suggest that BPAF may act as a developmental toxicant capable of persistently disrupting the immune–metabolic axis through epigenomic mechanisms, highlighting the need for careful re-evaluation of its use as a BPA substitute in consumer products.

## 1. Introduction

Bisphenol analogs (BPs), typical endocrine-disrupting chemicals, have been widely used in the manufacture of polycarbonate and epoxy resin [1]. Given BPA’s well-documented neurobehavioral, reproductive and developmental toxicity [2], industry has increasingly substituted it with other alternatives, including BPAF [3]. BPAF is now released from industrial products into air, water, and food, leading to widespread human exposure [4]. Recent studies reported BPAF was detected across diverse matrices, including surface waters [5], sewage, soils, indoor dust [6], and food [7], as well as in human urine [8] and blood [9,10]. Surface water contamination reached 140 ng/L in Lake Taihu [11], while sediment levels in Hangzhou Bay (2009.8 ng/g) far exceeded those of BPA (42.8 ng/g) [12]. A nationwide survey reported that the detection rate of BPAF in indoor dust was 30.1% in China, with an average content of 0.704 ng/g [13]. In South China, 75% of inhabitants’ urine contained BPAF with the median concentration at 0.15 μg/L [14]. BPAF was also detected in maternal plasma (9–168 pg/g), cord blood, placental tissue, and breast milk (mean 0.092 ng/mL), with evidence suggesting potential developmental impacts through lactational transfer [15,16]. These reports, coupled with BPAF’s stronger bioaccumulation potential and environmental persistence exceeding that of BPA, raise significant concerns about its adverse health effects [17,18].

Growing evidence links environmental chemical exposure to hepatic metabolic dysfunction and pathogenesis of metabolic dysfunction-associated steatotic liver disease (MASLD) through multiple mechanisms [19,20,21]. Epidemiological studies demonstrated significant associations between BPAF exposure and metabolic disorders, including positive serum correlations with hyperglycemia [22], elevated urinary levels in type 2 diabetes [23], and increased gestational diabetes risk [24]. During hepatic steatosis, abnormal lipid deposition often occurs simultaneously with insulin resistance and is associated with disruption of systemic immunity homeostasis [25]. Indeed, BPAF was reported to have the capacity to promote adipogenesis, lipid accumulation, and inflammatory signaling at environmentally relevant doses [26]. A couple of studies showed that BPAF exposure during critical developmental periods disrupted immune homeostasis, altering immune cell populations and macrophage function while activating pro-inflammatory pathways [27,28,29]. However, its capacity to induce intergenerational metabolic dysfunction and underlying molecular mechanisms remains unknown.

Epigenetic regulations include non-coding RNAs, DNA methylation and histone modification, which are master regulators of gene expression that orchestrate a series of biological processes, including immune and metabolic homeostasis [30,31,32]. The interactions between them form a dynamic regulatory network where enhancer activation and transcription factor binding reciprocally influence each other to maintain physiological balance. Numerous studies have reported that exposure to chemicals could induce widespread epigenetic dysregulation, altering histone marks (e.g., H3K27ac) at metabolism-related gene loci [33,34,35]. Such perturbations disrupted the delicate equilibrium between nuclear receptors (PPARs and LXRs) and immune transcription factors (CEBPs and NF-κB), leading to transcriptional reprogramming and metabolic dysfunction [36,37]. Liver is an important target organ of the metabolism of bisphenols in vivo [38] and plays a crucial role in the occurrence and development of metabolic syndrome [39]. Its epigenetic landscape is susceptible to environmental insults, with chemical-induced epigenetic changes capable of simultaneously impairing lipid homeostasis and inflammatory responses.

The multi-omics strategy provides a comprehensive insight into liver toxicity caused by harmful environmental factors. Our study used metabolomics, transcriptomics and epigenomics to systematically evaluate intergenerational hepatotoxicity of BPAF. We hypothesized that BPAF reprogrammed the expression of genes associated with lipid metabolism and immune inflammation, predisposing to MASLD. Our study provides novel insights into BPAF-induced hepatotoxicity, further enhancing our knowledge of the epigenetic mechanisms by which perinatal BPAF exposure leads to MASLD-related symptoms in offspring.

## 2. Materials and Methods

### 2.1. Animals and Chemical Treatment

This work has received approval for research ethics from the Institutional Animal Care and Use Committee of Nanjing Medical University. Seven-week-old C57BL/6J mice were purchased from the Animal Core Facility of Nanjing Medical University and housed in a specific-pathogen-free (SPF) barrier system with free access to drinking water and food as previously described [40]. BPAF was purchased from Aladdin (Shanghai, China), dissolved in dimethyl sulfoxide (DMSO) (Sigma-Aldrich, St. Louis, MO, USA) and stored in glass vials. After acclimation, dams were randomly assigned to receive BPAF (0, 0.04, 0.4 and 4 mg/kg·bw/day) from pre-mating to delivery in order to capture both germline and somatic programming effects. A total of 8 dams per group were initially mated. Of these, 7 control dams and 6, 6, and 4 dams in the low-, medium-, and high-dose BPAF groups, respectively, achieved successful pregnancy and delivered live litters. For each experimental endpoint, offspring were randomly selected from the pool of available pups within each treatment group, with efforts made to balance sex and litter representation. The BPAF exposure period spans the epigenetic reprogramming windows of zygote cleavage (GD 0.5~3.5) and hepatic progenitor cell differentiation (GD 12.5~postnatal). The selected doses (0.04~4 mg/kg·bw/day) reflect human-relevant exposure: 0.04 mg/kg aligns with urinary BPAF levels in pregnant women (median: 0.15 µg/L) [14,16], while 4 mg/kg approximates 10% of EPA’s NOAEL (the No Observed Adverse Effect Level established by the U.S. Environmental Protection Agency) for BPA [41].

Male and female mice were mated, and the presence of a vaginal plug the following morning was designated gestational day (GD) 0.5. Body weight and blood glucose of offspring were measured on postnatal day 7. Thereafter, body weight was recorded weekly. At seven weeks of age, offspring underwent glucose tolerance and insulin tolerance tests. A subset from each group was euthanized: serum was collected for lipid profiling, spleens for immunoassays, and livers were tested for metabolomics, RNA-seq, and CUT&Tag. The remaining animals were fed either a normal or 60% of high-fat diet (Xietong, Nanjing, China, XTM01-002) for an additional two months to assess long-term changes in hepatic metabolism. Details of the concise overview of sample allocation across assays can be found in Appendix A.

### 2.2. Glucose Tolerance Test and Insulin Tolerance Test

For the glucose tolerance test, mice were fasted overnight (9 pm to 9 am) and weighed before testing. A baseline blood glucose measurement was taken from a tail vein sample. Each mouse then received an intraperitoneal injection of D-glucose (2 g/kg body weight), and blood glucose was recorded at 15 min, 30 min, 60 min, 90 min and 120 min post-injection. For the insulin tolerance test, mice were fasted from 8 am to 2 pm and weighed before testing. Insulin (0.75 U/kg) was administered via intraperitoneal injection, and glucose levels were measured at 15 min, 30 min, 60 min, 90 min and 120 min. The resulting glucose–time profile was plotted, and the area under the curve (AUC) was calculated.

### 2.3. H&E Staining

The liver, brown fat and white adipose tissues were fixed in 4% paraformaldehyde for 24 h, gradient dehydrated with alcohol and embedded in paraffin. The paraffin-embedded blocks were cut into 5 µm sections and stained with hematoxylin and eosin (H&E). Images were captured and analyzed using a panoramic MIDI digital camera (Diagnostic Instruments, 3DHISTECH, Budapest, Hungary).

### 2.4. Oil Red O Staining

Frozen liver tissue was embedded in OCT (optimal cutting temperature compound). Tissue blocks were cut into 10 μm slices, fixed in glacial acetic acid–methanol for 1 min, washed with PBS and stained with Oil Red O solution for 30 min. Then, the liver slices were differentiated in 60% isopropyl alcohol for 5 min to remove the excess dye, stained in hematoxylin for 30 s and sealed with 60% glycerin. Images were captured using a panoramic MIDI digital camera and analyzed through Image J v1.54p software.

### 2.5. Transcriptomic Analysis

Total RNA was extracted using the Trizol kit (Thermo Fisher Scientific, Waltham, MA, USA). The concentration and integrity were measured using the RNA Nano 6000 Assay Kit of the Bioanalyzer 2100 system (Agilent Technologies, Santa Clara, CA, USA). The mRNA was purified from total RNA using poly-T oligo-attached magnetic beads. Sequencing libraries were generated by NEBNext UltraTM RNA Library Prep Kit for Illumina (NEB, Ipswich, MA, USA), assessed by Qubit2.0 Fluorometer and Agilent Bioanalyzer 2100 system, and sequenced on an Illumina Novaseq platform and 150 bp paired-end reads. Raw reads were removed as low-quality reads and trimmed adapter sequences were removed using fastp [42]. The cleaned reads were then aligned to the mm10 reference genome using STAR [43], and quantified by featureCounts [44]. Differentially expressed gene analysis was performed using pyDeseq2 [45] with an FDR threshold of <0.05. Gene Ontology (GO), Kyoto Encyclopedia of Genes and Genomes (KEGG) pathway, and gene set enrichment analyses (GSEAs) were conducted using the GSEApy v1.1.11 software [46].

### 2.6. Metabolomics Analysis

Liver tissues were homogenized and sonicated in an ice-water bath, then incubated for 1 h and centrifuged at 4 °C (12,000 rpm, 15 min). About 0.5 mL of the supernatant was transferred to a fresh glass vial for metabolome analysis (BioTree, Shanghai, China). The non-target metabolome was performed using a UHPLC system (Vanquish, Thermo Fisher Scientific) with a UPLC BEH Amide column (2.1 mm × 100 mm, 1.7 μm) coupled to a Q Exactive HFX mass spectrometer (Orbitrap MS, Thermo, Waltham, MA, USA).

We utilized Partial Least Squares Discriminant Analysis (PLS-DA) from the ropls v1.42.0 R package to illustrate group separation and calculated the Variable Importance in Projection (VIP) value for each metabolite [47]. *p*-values between groups were calculated using a *t* test, and differentially expressed metabolites (DEMs) were determined by *p* < 0.05, VIP > 1, and Fold Change > 1.5. The KEGG enrichment analysis was performed using MetaboAnalyst (Version 5.0) [48].

### 2.7. Cleavage Under Targets and Tagmentation (CUT&Tag)

H3K27ac CUT&Tag was performed as previously described with minor modifications [49]. In brief, we lysed 1 × 10^5^ liver cells in an ice-cold buffer containing 10 mM KCl, 20 mM HEPES, 0.5 mM spermidine, 20% glycerol, 0.1% Triton-X100 and 1× Protease Inhibitor on ice for 10 min, followed by light crosslinking with 0.01% formaldehyde to fix nuclei for 2 min at room temperature before quenching with 75 mM glycine. Then, nuclei were transferred into a wash buffer containing 150 mM NaCl, 20 mM HEPES, 0.5 mM spermidine, 1× protease inhibitor and bound to Concanavalin A-Coated Magnetic Beads (N251, NoVoNGS, Beijing, China). The mixture was incubated with 0.5 μL H3K27ac antibody (ab4729, Abcam, Waltham, MA, USA) at 4 °C overnight in a 50 μL antibody buffer (2 mM EDTA and 0.1% (wt/vol) BSA). After washing, the nuclei were mixed with pG-Tn5 in digitonin buffer (0.5 mM spermidine, 20 mM HEPES, 300 mM NaCl, 0.01% digitonin, and 1× protease inhibitors) at room temperature for 1 h. The cells were resuspended in a 300 μL fragmentation buffer containing digitonin buffer with 10 mM MgCl_2_ and incubated 37 °C for 1 h. In total, 10 µL 0.5 M EDTA, 2.5 µL 20 mg/mL Proteinase K and 3 µL 10% SDS were added to each sample and incubated for 1 h at 50 °C to stop fragmentation and solubilize DNA fragments. Finally, DNA was extracted, amplified and sequenced on NovaSeq 6000 (NovoGene, Tianjin, China). Raw reads (*n* = 2 per group) were filtered using Fastp and mapped to the mm10 with bowtie2 [50]. Unique alignments underwent deduplication using Picard MarkDuplicates v2.18.16 (https://broadinstitute.github.io/picard/ accessed on 20 May 2025). Peak calling and differential peak analysis were performed using Sicer [51]. Specifically, peaks were called with the *sicer* command using the following parameters: window_size = 200, gap_size = 600, and FDR = 0.01. Differential peak analysis was conducted using *sicer_df* with an FDR threshold of 0.01. Motif analysis was conducted with HOMER [52]. Enrichment analysis was carried out using GREAT v4.0.4 tools [53], and peak annotation was performed using the ChipSeeker v1.36.0 package [54]. Heatmaps and profiles for normalized CUT&Tag data were generated using the bamCoverage function from the DeepTools v3.5.6 [55].

### 2.8. Flow Cytometry

The spleen tissue was homogenized and added to the ice-cold PBS containing 1% FBS (Gibco, Waltham, MA, USA) and 1% EDTA (Sigma-Aldrich, USA). Red blood cells were lysed with ammonium–chloride–potassium (ACK) lysis buffer. Cell viability was determined by Trypan blue (required >95% viable cells). As described in previous work [56], after cell-surface staining, cells were fixed, permeabilized and specifically labeled with the corresponding antibodies. CD3-Percp-cy5.5, CD4-FITC and CD8-PE antibodies were used for CD3 T, CD4 T and CD8 T cell characterization, respectively. CD3-Percp-cy5.5 and NK1.1-BV421 antibodies were used for NK T and NK cell characterization. CD4-FITC, CD25-APC and Foxp3 antibodies were used for Treg cells characterization. CD4-FITC, CD25-APC, CXCR5-PE/Cy7 and PD1-PE antibodies were used for Tfh cells characterization. Moreover, macrophages were marked with CD11b-FITC, F4/80-PE/Cy7, and CD86-PE antibodies. All the above antibodies are from eBioscience, San Diego, CA, USA. Events were subsequently acquired using an FACSVerse flow cytometer (BD) (Becton, Dickinson and Company, Franklin Lakes, NJ, USA) and analyzed with FlowJo v10 software (Treestar).

### 2.9. Statistical Analysis

All statistical analyses were conducted using two-tailed Student’s *t* test and one-way ANOVA using Prism8 and RStudio v4.1.4. All the data were presented as the means  ±  SEM. The level of significance was set at * *p*  <  0.05, ** *p* < 0.01.

## 3. Results

### 3.1. Perinatal BPAF Exposure Altered Body Weight and Glucose Metabolism in Offspring

**Figure 1:**
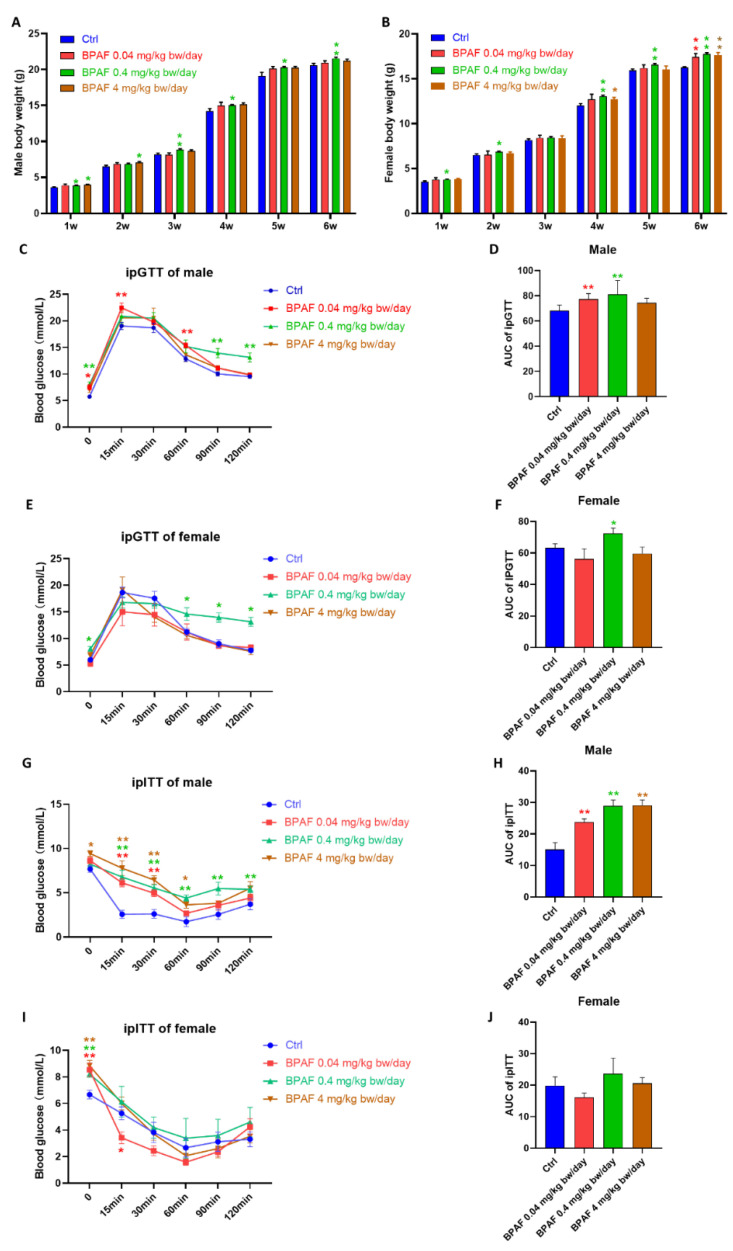
Perinatal BPAF exposure altered body weight and blood glucose levels in offspring. Body weight in (**A**) male and (**B**) female offspring after dams were exposed to different doses of BPAF (*n* = 8 per group). Blood glucose levels (**C**) and the AUC (**D**) for ipGTT of male offspring (*n* = 8 per group). Blood glucose levels (**E**) and the AUC (**F**) for ipGTT of female offspring (*n* = 8 per group). Blood glucose levels (**G**) and the AUC (**H**) for ipITT of male offspring (*n* = 8 per group). Blood glucose levels (**I**) and the AUC (**J**) for ipITT of female offspring (*n* = 8 per group). Colors represent different treatment groups: blue for control, red for BPAF 0.04 mg/kg, green for BPAF 0.4 mg/kg, and brown for BPAF 4 mg/kg. Significance was calculated using a non-paired two-tailed Student’s *t* test or one-way ANOVA analysis; values are presented as mean ± SEM. * *p* < 0.05; ** *p* < 0.01.

**Figure A1:**
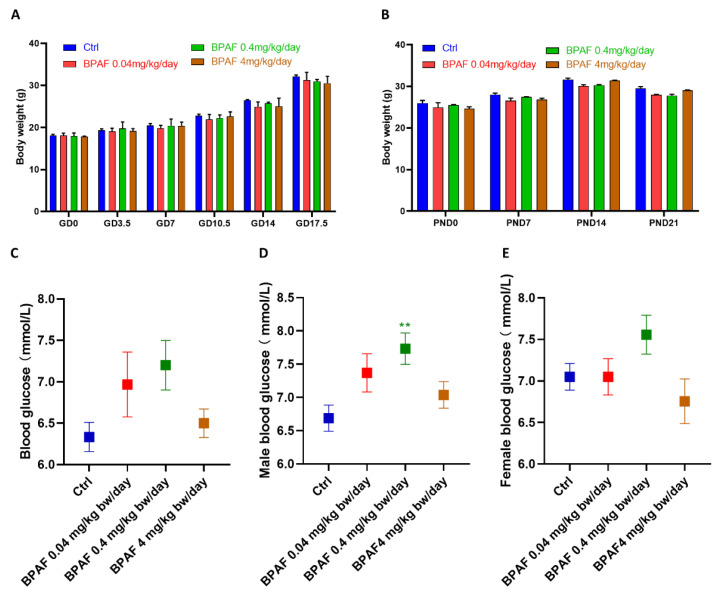
Perinatal BPAF exposure altered body weight and glucose metabolism in maternal dams and offspring. Body weight of dams during (**A**) pregnancy and (**B**) lactation period after exposure to different doses of BPAF. (**C**) Blood glucose levels of dams after exposure to different doses of BPAF. Blood glucose levels in 7-day-old (**D**) male offspring and (**E**) female offspring. Significance was calculated using one-way ANOVA analysis; values are presented as mean ± SEM. ** *p* < 0.01.

To assess the impact of BPAF exposure on maternal and offspring health, we monitored body weight and blood glucose in both groups (Figure A1A,B). We observed that offspring of BPAF-exposed mothers exhibited increased body weight (Figure 1A,B) and blood glucose levels (Figure A1C–E) relative to controls. The most pronounced effect on body weight was observed in the group exposed to the BPAF at 0.4 mg/kg·bw/day, regardless of gender.

To determine whether prenatal BPAF exposure induces dysglycemia in offspring, we performed the intraperitoneal glucose tolerance test (ipGTT) and intraperitoneal insulin tolerance test (ipITT). Perinatal BPAF exposure caused significant differences in ipGTT in offspring regardless of gender (Figure 1C–F). For the ipITT, we only observed significant differences in male offspring (Figure 1G–J). These findings indicate that perinatal BPAF exposure reduces insulin sensitivity in offspring, especially in male offspring. Exposure to 0.4 mg/kg/day exhibited the most substantial effects on body weight and glucose metabolism, especially in male offspring. Moreover, after exposure to BPAF (0.4 mg/kg/day), we detected BPAF in blood concentration of dams (0.25~0.92 ng/mL), which was close to the content of BPAF (0.092~0.58 ng/mL) in breast milk reported by Jin et al. [16]. Therefore, we focused on this dose (0.4 mg/kg/day) in subsequent analyses of molecular mechanisms.

### 3.2. Perinatal BPAF Exposure Caused Hepatic Lipid Accumulation and Adipose Hypotrophy

**Figure 2:**
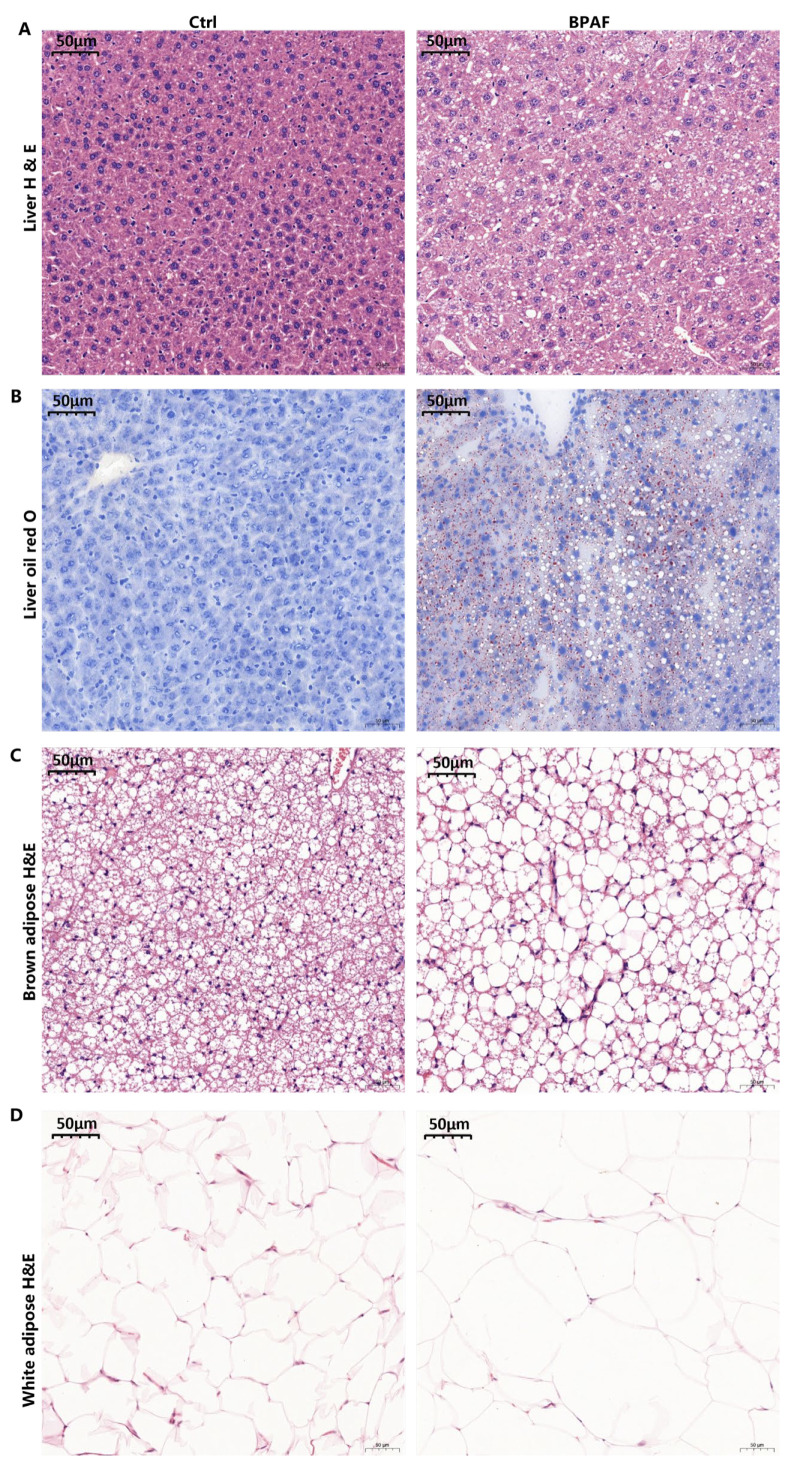
Perinatal BPAF exposure caused hepatic lipid accumulation and adipose hypotrophy. (**A**) H&E staining and (**B**) Oil Red O staining of liver tissues in different groups. H&E staining of (**C**) white adipose tissues and (**D**) brown adipose in different groups. Scale bar, 50 μm.

To evaluate lipid deposition in liver tissue, we conducted Oil Red O and H&E staining on liver sections. BPAF exposure led to increased lipid accumulation in hepatocytes (Figure 2A). In the control group, liver tissue architecture was intact, with hepatocytes displaying normal organization, clearly defined nuclei and cytoplasm, and no signs of inflammation, fibrosis, or steatosis. In contrast, the BPAF group exhibited significant histological alterations, including disrupted morphology, disorganized hepatic lobules, cytoplasmic vacuolization, and mild-to-moderate steatosis, indicative of metabolic disturbances. Oil Red O staining further corroborated these findings, revealing sparse liver morphology, clearly visible fat droplets, and numerous differently shaped vacuoles after BPAF exposure (Figure 2B). These results indicate that perinatal BPAF exposure significantly disrupts lipid metabolism, leading to excessive lipid accumulation in the liver.

Moreover, we observed notable changes in adipose tissue structure, including enlarged adipocytes and irregularly shaped cells (Figure 2C) in the BPAF group. Brown adipose tissue also showed significant alterations, characterized by a loose and disordered arrangement of adipocytes, numerous vacuoles, and an increase in cell diameter (Figure 2D). Collectively, these findings suggest that perinatal BPAF exposure induces hypotrophy of white adipose tissue and whitening of brown adipose tissue in offspring mice.

**Figure A2:**
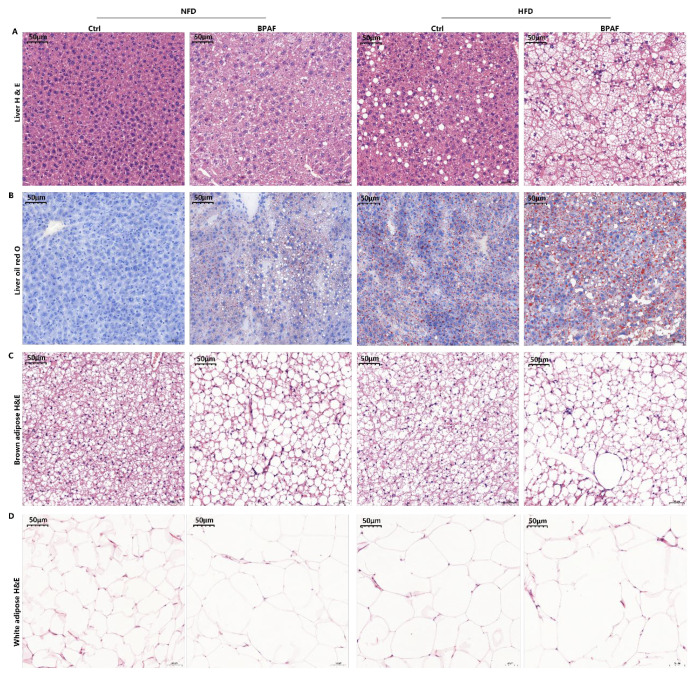
HFD worsened the lipid deposition in offspring after perinatal BPAF exposure. (**A**) H&E staining and (**B**) Oil Red O staining of liver tissues in different groups. H&E staining of (**C**) white fat tissues and (**D**) brown fat in different groups. Scale bar, 50 μm.

To understand whether perinatal BPAF exposure predisposes offspring to MASLD, we fed the offspring mice on HFD for another 8 weeks. As anticipated, HFD worsened lipid deposition in offspring from the BPAF exposure group, characterized by sparse tissue structures, an obvious increase in fat droplets in the liver, and an obvious increase in fat vacuoles in white fat and brown fat (Figure A2).

### 3.3. Perinatal BPAF Exposure Altered the Inflammatory Profile of the Spleen in Offspring

**Figure 3:**
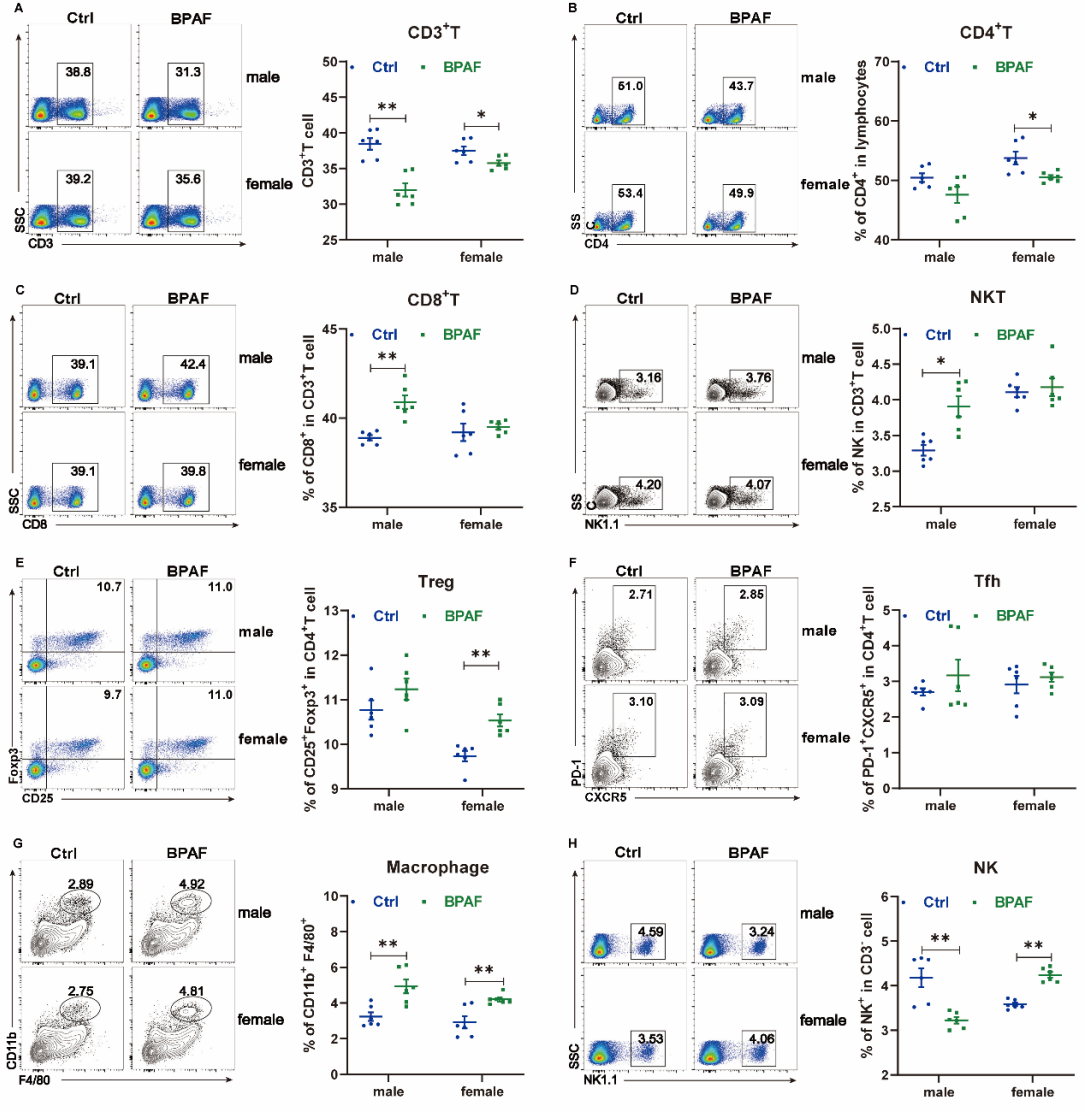
Perinatal BPAF exposure altered spleen inflammatory status in offspring. Flow cytometric analysis of (**A**) CD3+ T cells, (**B**) CD4+ T cells, (**C**) CD8+ T cells, (**D**) NKT cells, (**E**) Treg cells, (**F**) Tfh cells, (**G**) macrophages and (**H**) NK cells, (*n* = 6–8 per group). Significance was calculated using non-paired two-tailed Student’s *t* test; values are presented as mean ± SEM. * *p* < 0.05; ** *p* < 0.01.

Given that metabolic syndrome often triggers inflammation, we investigated whether perinatal BPAF exposure alters immune characteristics in offspring. We observed that perinatal BPAF exposure led to immune remodeling, as evidenced by significant reductions in the proportions of CD3^+^ and CD4^+^ T cells in the spleens of offspring (Figure 3A,B). Sex-specific changes were noted in cytotoxic lineages: male offspring exhibited a marked increase in CD8^+^ T and NKT cells, while female offspring showed no significant changes (Figure 3C,D). Additionally, maternal BPAF exposure resulted in consistent trends across all offspring regarding CD4^+^ T cell subsets, with significant increases in regulatory T (Treg) cells and a slight rise in T follicular helper (Tfh) cells (Figure 3E,F). Moreover, the percentage of macrophages was significantly elevated in both male and female offspring (Figure 3G). We observed opposite trends of changed NK cells between male and female offspring (Figure 3H).

### 3.4. Perinatal BPAF Exposure Changed Hepatic Metabolome in Offspring

**Figure 4:**
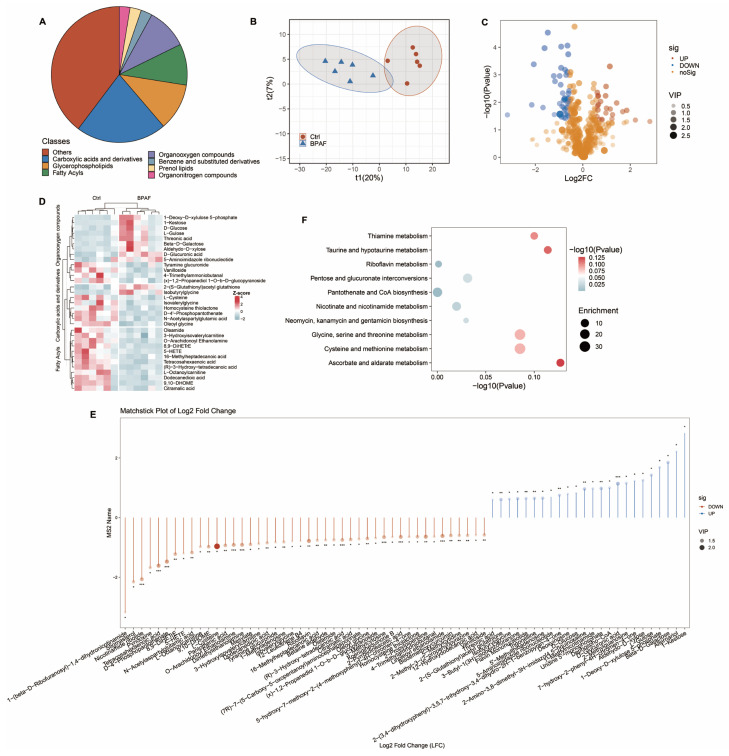
Perinatal BPAF exposure changed hepatic metabolome in offspring. (**A**) Categorization of metabolites detected by LC-MS/MS (*n* = 6 per group). (**B**) PLS-DA plot of metabolites and (**C**) Volcano plot of differentially expressed metabolites (DEMs) between the control and BPAF exposure group, defined by VIP > 1, *p* < 0.05, and Fold Change > 1.5. Dot size represent VIP score. (**D**) Heatmap plot of the predominant classes among these DEMs. (**E**) Matchstick analysis plot of DEMs between the control and BPAF group. Significance was calculated using a non-paired two-tailed Student’s *t* test (* *p* <0.05, ** *p* < 0.01, *** *p* < 0.001). Dot size and color shade represent VIP score. (**F**) Dot plot displaying the KEGG enrichment analysis results of DEMs.

To investigate how BPAF disrupts liver metabolites in offspring, we performed untargeted metabolomic analysis using LC-MS/MS (Appendix A). We identified 738 metabolites classified into 94 distinct categories, with the largest contributions originating from carboxylic acids and derivatives, followed by glycerophospholipids, organooxygen compounds, and fatty acyls (Figure 4A). Partial least squares discriminant analysis (PLS-DA) revealed distinct metabolomic profiles between the control and BPAF-treated groups (Figure 4B). PLS-DA was performed on 738 metabolites from 16 samples (*n* = 6 per group). The model used four latent components and explained 98.9% of the class variance (R^2^Y = 0.989) with a predictive ability of Q^2^ = 0.774. Permutation testing (200 iterations) yielded *p* = 0.005 for both R^2^Y and Q^2^, indicating significant separation between groups. A comparison of BPAF-exposed and control groups identified 68 differentially expressed metabolites (DEMs), with 24 metabolites exhibiting increases and 44 metabolites displaying decreases (VIP > 1, *p*-value < 0.05, Fold Change > 1.5; Figure 4C, Table A1). The predominant classes among these DEMs included 13 organooxygen compounds, 12 fatty acyls, and 8 carboxylic acids and derivatives (Figure 4D). BPAF exposure significantly reduced D-4′-Phosphopantothenate and tetracosahexaenoic acid while elevating D-Glucuronic acid and Avenalumin III in male offspring (Figure 4E). KEGG pathway analysis of DEMs revealed enrichment in pathways related to pantothenate and CoA biosynthesis, glycerophospholipid metabolism, riboflavin metabolism and glycine, serine, and threonine metabolism following maternal BPAF exposure (Figure 4F).

### 3.5. Perinatal BPAF Exposure Reprogramed Hepatic Transcriptome in Offspring

**Figure 5:**
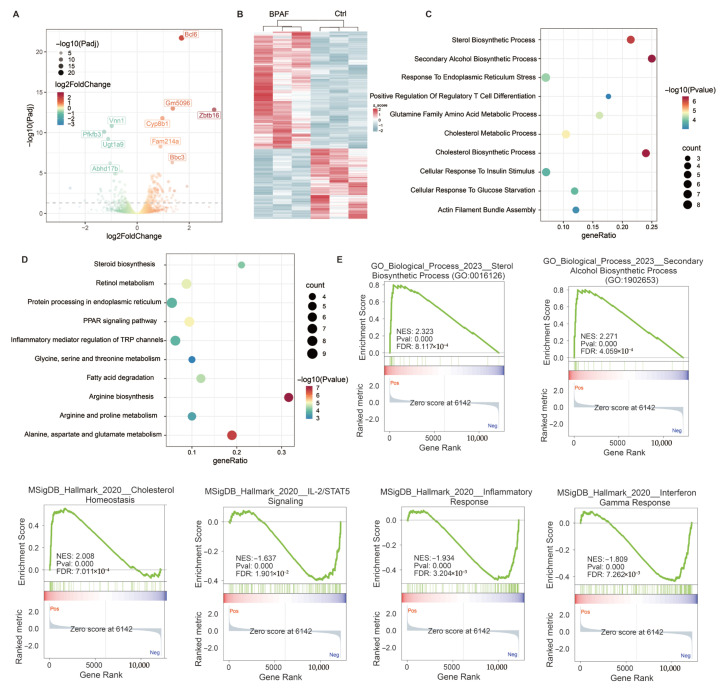
Perinatal BPAF exposure reprogrammed hepatic transcriptome in offspring. (**A**) Volcano plot of differentially expressed genes (DEGs) and (**B**) heatmap of DEGs between the control and BPAF exposure group, defined by adjusted *p*-value < 0.05. (**C**) Dot plot of the GO enrichment analysis results of DEGs. (**D**) Dot plot of the KEGG enrichment analysis results of DEGs. (**E**) Gene sets enrichment analysis (GSEA) of genes ranked by log_2_ Fold Change using the MSigDB database.

**Figure A3:**
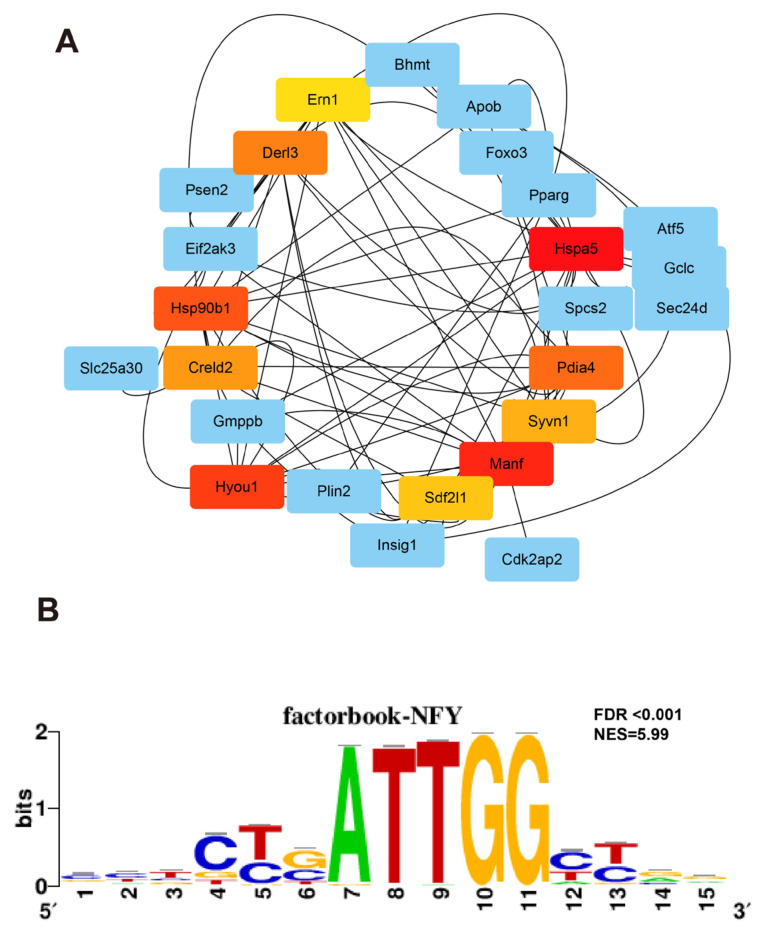
Hub genes and transcription factors associated with BPAF exposure. (**A**) The hub genes identified from DEGs following BPAF exposure, ranked by Maximal Clique Centrality (MCC). Color intensity corresponds to the MCC score, with darker red indicating higher centrality. (**B**) DNA binding motif of the most significantly enriched transcription factor (Nfya; NES = 5.99, FDR < 0.001) in the promoters of DEGs, as predicted by iRegulon v1.2.

To elucidate the molecular mechanisms of BPAF-induced toxicity in offspring, we performed RNA-seq and identified 148 upregulated and 89 downregulated genes (FDR < 0.05, Figure 5A,B, Figure A3). GO analysis of DEGs revealed significant alterations in lipid and amino acid metabolic pathways, including cholesterol/sterol biosynthesis and glutamine metabolism, along with PPAR signaling and arginine/alanine/aspartate and glutamate metabolism in KEGG analysis (Figure 5C,D). Gene set enrichment analysis demonstrated concurrent downregulation of immune pathways (interferon gamma response, IL2/STAT5 signaling, and inflammatory response) and upregulation of metabolic processes (sterol/cholesterol biosynthesis), indicating that BPAF exposure induces hepatic metabolic dysfunction while suppressing immune responses in offspring (Figure 5E). Collectively, these results suggest that BPAF exposure significantly disrupts immune processes, in particular the inflammatory response, inducing hepatic lipid metabolic and amino acid dysfunction.

We reconstructed a protein–protein interaction (PPI) network using the identified DEGs and identified top hub genes based on Maximal Clique Centrality (MCC). As a result, we identified several high-ranking hub genes, including Hspa5, Ern1, and Manf. These genes are not only central to the unfolded protein response but also directly implicated in metabolic regulation (Figure A3A). Specifically, Hspa5 is an endoplasmic reticulum chaperone that regulates cell metabolism, particularly lipid metabolism. Furthermore, we predicted the transcription factors (TFs) linked to DEGs using the iRegulon plugin in Cytoscape v3.10.2. We found that the most significantly enriched transcription factor Nfya (NES = 5.99, FDR < 0.001) regulated the 95 DEGs, including Hspa5, Manf and Creld2 genes (Figure A3B). Recent evidence has demonstrated that Hspa5 regulates pre-RNA alternative splicing, stability, or translation and affects target proteins via binding to lncRNA and mRNA linked to MASLD ([57]). Together, these findings suggest that BPAF exposure may interfere with transcription factor binding, thereby altering the expression of downstream genes.

### 3.6. Joint Analysis of the Hepatic Transcriptome and Metabolome

**Figure 6:**
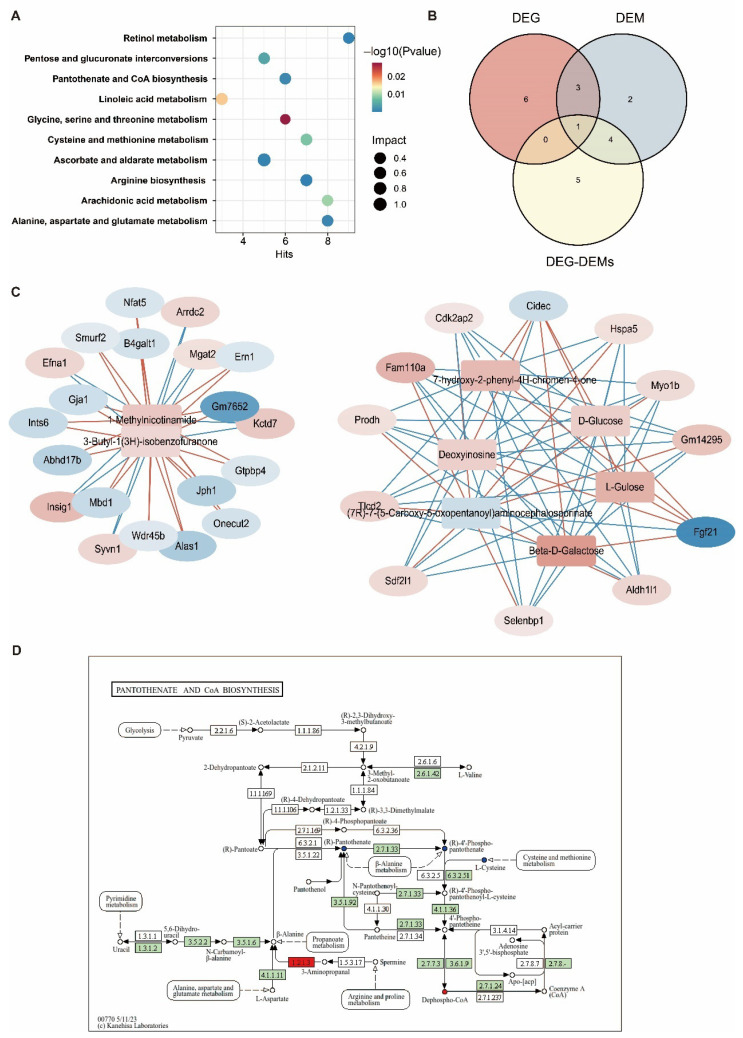
Integrated analysis of the hepatic transcriptome and metabolome. (**A**) Dot plot of the KEGG enrichment analysis results of DEG-DEMs. (**B**) Venn diagram of the top 10 enriched KEGG pathways between DEGs, DEMs and DEG-GEMs. (**C**) The DEG-GEMs network. Rectangles and circles represent DEMs and DEGs, respectively. Red and blue colors represent upregulated and downregulated DEMs or DEGs in the BPAF group. (**D**) Schematic representation of Pantothenate and CoA biosynthesis pathways associated with differentially enriched metabolites and genes. Metabolites/genes increased in the BPAF group are indicated by red-filled modules and metabolites/genes decreased in the BPF group are marked by green-filled modules.

Integrated transcriptomic–metabolomic analysis revealed BPAF-induced hepatic adaptations through coordinated pathway alterations, with retinol metabolism, arginine biosynthesis, and pantothenate/CoA biosynthesis being the most significantly enriched (Figure 6A). The co-occurrence of glycine, serine and threonine metabolism in the top 10 KEGG pathways for DEGs, DEMs and DEG-DEMs suggested that BPAF exposure disrupted the amino acids at both hepatic transcriptome and metabolome levels (Figure 6B). We further performed DEG-DEM Spearman correlation analysis to construct the metabolite–gene network. The network consisted of 145 nodes (37 metabolites, 108 genes) and 221 edges. Out of a total number of edges, 40.7% (80) were positive correlations and 59.3% (131) were negative correlations. The metabolite showing the highest degree was 3-Butyl-1(3H)-isobenzofuranone and 1-Methylnicotinamide, with significant correlations to 20 different genes. In contrast, the genes (*Sdf2l1*, *Cdk2ap2*, *Fgf21*, etc.) had the highest connectivity with six metabolites (Figure 6C). Furthermore, the enrichment of four DEMs (Dephospho-CoA, Pantothenic acid, D-4′-Phosphopantothenate, and L-Cysteine) and two DEGs (*Vnn1*, *Aldh1b1*) in the Pantothenate and CoA biosynthesis pathway underscores its potential role in mediating hepatic responses to BPAF exposure (Figure 6D). Together, these findings highlight the complex interplay between gene expression and metabolic changes in response to BPAF exposure, indicating potential key pathways and molecules involved in hepatic adaptation.

### 3.7. Perinatal BPAF Exposure Remodels Hepatic Enhancer Landscape to Regulate Gene Expressions in Offspring

**Figure 7:**
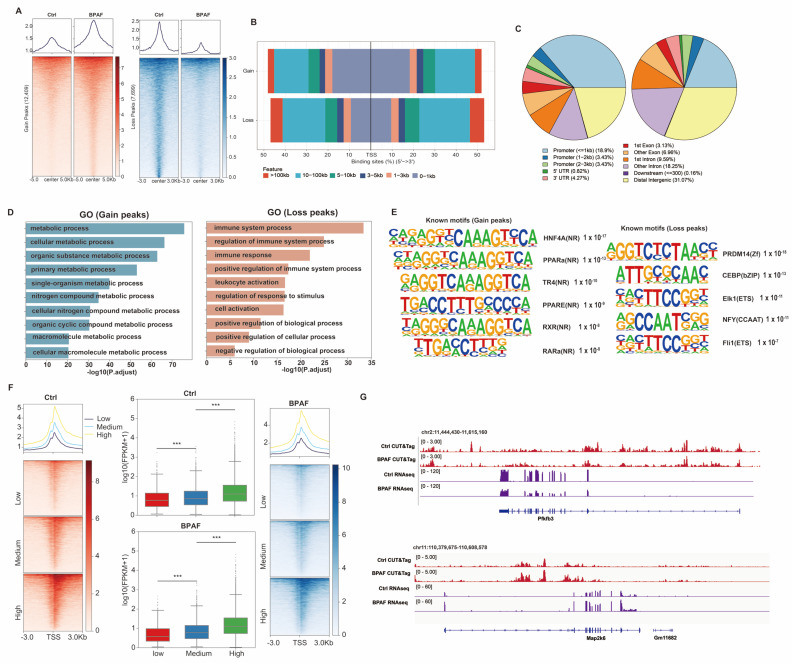
Perinatal BPAF exposure remodeled hepatic enhancer landscape to regulate gene expressions in offspring. (**A**) Metagene heatmap plot of BPAF induced differential enhancer activity regions in control and BPAF groups (*n* = 2 per group). (**B**,**C**) Genomic annotation of differential enhancer activity regions using the ChIPseeker. (**D**) Bar plot of the GO enrichment analysis of gain and loss regions using the GREAT webserver. (**E**) Motifs enriched at gain and loss H3K27ac CUT&Tag regions. (**F**) Metagene plot and bar plot show the correlation between H3K27ac CUT&Tag signal at TSS and gene expression in the control and BPAF exposure groups. (**G**) Example loci of differential H3K27ac CUT&Tag regions near gene TSSs. Significance was calculated using the Wilcoxon rank-sum test. *** *p* < 0.001.

Gene expression is regulated by regulatory elements, such as enhancers. Here, to understand why the mechanism of BPAF caused gene expression changes, we profiled the hepatic active enhancer landscape using CUT&Tag. We obtained a total of 19.3 Gb of raw sequencing data, with an average mapping rate of 81% and a FRiP (fraction of reads in peaks) of 12.7%, indicating high-quality enrichment for H3K27ac (Table A1). CUT&Tag analysis of H3K27ac (active enhancer marker) in offspring liver revealed that BPAF exposure-induced genome-wide changes, with 36.8% (20,188) loci showing differential acetylation (7699 lost and 12,409 gained peaks, Figure 7A). Gain peaks preferentially localized near-transcription start sites (TSSs) compared to loss peaks, while genomic annotation showed distinct distribution patterns between them (Figure 7B). Functional analysis demonstrated that loss peaks were enriched for immune-related pathways (leukocyte activation and immune response), whereas gain peaks were associated with metabolic processes (cellular/organic metabolism and nitrogen compound metabolism) (Figure 7D). Motif analysis identified immune-related transcription factors (CEBP and Elk1) in loss regions and nuclear receptor family metabolic regulators (HNF4A, PPARA, and RXR) in gain regions (Figure 7E).

In addition, the H3K27ac signal was reported to be correlated with gene expression. We divided genes into low, medium, or high groups according to their expression levels and then categorized matched H3K27ac CUT&Tag data to calculate the enrichment in TSS regions. The boxplot revealed that highly expressed genes possessed more H3K27ac in TSS in both groups (Figure 7F). Example loci depicting differential H3K27ac enrichments in TSS and gene expression are presented in Figure 7G. These epigenetic alterations correlated with transcriptional shifts toward disturbed inflammatory states and metabolic dysregulation, indicating that BPAF exposure reprograms the hepatic epigenomic landscape by suppressing immune-related enhancers while activating metabolic enhancer regions, ultimately leading to metabolic dysregulation.

## 4. Discussion

BPAF has raised emerging health concerns due to its potential toxicity. Although extensive research has documented BPAF’s multi-organ toxicity, including effects on the endocrine, reproductive, and nervous systems [2,28,29], its intergenerational metabolic consequences remain poorly understood. Given the liver’s central role in metabolic regulation and its vulnerability to environmental toxicants, we focused on hepatic outcomes in offspring following perinatal BPAF exposure. We observed that perinatal BPAF exposure disrupted hepatic lipid metabolism through coordinated alterations in gene expression, enhancer activity, and metabolic reprogramming. Furthermore, this metabolic dysregulation was associated with perturbations in immune–inflammatory gene networks and was exacerbated by HFD exposure. These findings highlight the critical role of immune–inflammatory crosstalk in BPAF-induced hepatic lipid accumulation, offering novel mechanistic insights into the pathogenesis of MASLD and underscoring the urgent need to reassess BPAF’s safety during developmental windows.

Our results are consistent with prior studies demonstrating BPAF-mediated induction of systemic inflammation [28,40] and disruption of arachidonic acid metabolism—a key pathway involved in the production of inflammatory mediators such as prostaglandins and leukotrienes [58]. Transcriptomic profiling in our study identified significant enrichment of pathways related to interferon gamma response, IL2/STAT5 signaling, and inflammatory response, while enhancer analysis revealed activation of immune-related processes, including immune system regulation and leukocyte activation. These coordinated molecular changes indicated that BPAF perturbed hepatic lipid homeostasis via immune-inflammatory mechanisms, aligning with well-established links between chronic inflammation and MASLD progression [25,59]. The integration of metabolic and immune alterations emphasizes the intricate interplay between immunity and metabolism in the context of BPAF exposure, suggesting that developmental exposure to this chemical may predispose offspring to metabolic liver diseases through a dual metabolic–immune axis.

Numerous studies demonstrated that pro-inflammatory cytokines (IL-6, TNF-α, IL-1β, and IFN-γ) played pivotal roles in initiating liver inflammation and promoting metabolic disorders, often preceding hepatic steatosis development [59,60]. Our findings reveal that prenatal BPAF exposure triggers a cascade of immunometabolic disturbances, characterized by inflammatory activation through cholesterol-mediated cytokine production [60], significant alterations in T cell populations and transcriptional reprogramming of both immune-related and metabolic-related gene networks. Particularly noteworthy was the observed disruption of arachidonic acid metabolism—a critical pathway generating inflammatory mediators like prostaglandins and leukotrienes [58]—which mechanistically links BPAF exposure to MASLD pathogenesis through cytokine-mediated metabolic reprogramming in offspring. These multi-level alterations collectively establish an inflammatory microenvironment conducive to hepatic metabolic dysfunction.

Our recent study highlights the crucial role of regulatory elements in coordinately regulating gene networks during disease pathogenesis [61]. BPAF exposure induced significant enhancer activation in offspring hepatocytes, characterized by TSS-proximal gain peaks corresponding to potential enhancers. These epigenetic alterations were functionally associated with differential occupancy of key transcription factors, including C/EBP family members and PPARs. Specifically, we observed BPAF-mediated dysregulation of C/EBPα, a bZIP transcription factor that orchestrates inflammatory responses through MAPK/NF-κB and JAK/STAT3 signaling [62]. We also observed perturbations in PPARα/γ expression, nuclear receptors that critically regulate hepatic β-oxidation and lipid metabolism [63]. BPAF was reported to exhibit the highest environmental persistence over the other bisphenol analogs due to its two trifluoromethyl (−CF3) groups [5]. The −CF3 groups of BPAF also confer epigenetic potency by affecting PPAR binding [64]. These findings demonstrate how BPAF exposure epigenetically reprograms both inflammatory and metabolic pathways through coordinated changes in enhancers and transcription factor activity, potentially creating a permissive environment for hepatic metabolic dysfunction.

In conclusion, our study suggests that BPAF is a persistent metabolic disruptor by crossing the placental barrier and inducing lasting gene-regulatory changes that persist beyond the exposure period. Future studies are needed to understand BPAF’s intergenerational effects in humans.

## Data Availability

The original contributions presented in this study are included in the article. Further inquiries can be directed to the corresponding authors.

## Appendix A

**Table A1:** Quality control metrics for CUTTag sequencing data.

	Total Bases (Gb)	Total Reads	Mapping Rate	FRIP (%)
Ctrl1	3.3	5,495,346	73.1	12.3
Ctrl2	4.5	7,500,946	78.6	14.1
BPAF1	6.8	11,277,323	85.1	12.9
BPAF2	4.7	7,857,515	87.2	11.8

FRIP: fraction of reads in peaks.
